# 

**Published:** 1946

**Authors:** 


### The Bristol
### Medico-Chirurgical Journal

A JOURNAL OF THE MEDICAL SCIENCES FOR THE
WEST OF ENGLAND AND SOUTH WALES

PUBLISHED UNDER THE AUSPICES OF
*THE BRISTOL MEDICO-CHIRURGICAL SOCIETY*

EDITOR
J. A. NIXON, C.M.G., M.D., F.R.C.P.
WITH WHOM ARE ASSOCIATED
A. RENDLE SHORT, M.D., B.S., B.Sc., F.R.C.S.
W. MELVILLE CAPPER, M.B., B.S., F.R.C.S.
G. E. F. SUTTON, M.C., M.D., B.S., M.R.C.P.
E. WATSON-WILLIAMS, M.C., Ch.M., F.R.C.S., Editorial Secretary

---

"Scire est nescire, nisi id me

Scire alius sciret"

---

*VOL. LXIII*

1946

BRISTOL: J. W. ARROWSMITH LTD.

### Contents of Vol. LXIII

Page

Nature and Science. Presidential Address. By Hugh H. Carleton, M.A., D.M., B.Ch., F.R.C.P. .. .. .. .. .. .. .. .. 1

The Diagnosis and Treatment of Rheumatoid Arthritis. By G. D. Kersley, M.D., F.R.C.P. .. .. .. .. .. .. .. .. .. 11

The Management of Peptic Ulcer. By Norman C. Tanner, M.B., Ch.B., F.R.C.S. .. .. .. .. .. .. .. .. .. .. 16

The British Red Cross in North-West Europe. By Lieut.-Col. K. M. Agnew, D.S.O., M.C., R.A. .. .. .. .. .. .. .. .. 31

The Diagnosis of Tumours of the Central Nervous System. By Diana J. Kinloch Beck, M.B., B.S., F.R.C.S. .. .. .. .. .. 65

Diagnosis of Tumours of the Central Nervous System. By Sir Hugh Cairns, D.M., F.R.C.S. .. .. .. .. .. .. .. .. .. 73

Dioscorides Phakas on the Detection of Iron in Blood, Urine and Sputum. By M. Nierenstein, D.Sc. .. .. .. .. .. .. .. 75

Inhalation of Food during Injection of Pentothal. By R. Hastings Moore, M.B., Ch.B., M.R.C.S., L.R.C.P., D.A. .. .. .. .. .. 82

The Thirty-third Long Fox Memorial Lecture : English Doctors from Smollett to Trollope. By E. Watson-Williams, Ch.M. .. .. .. .. 89

Symptomless Hyperpiesia in Dockyard Workers. By J. M. Naish, M.A., M.B., B.Ch., M.R.C.P.. .. .. .. .. .. .. .. 106

Pituitary Cachexia Treated with Corticotropic Hormone. By R. E. Hemphill, M.A., M.D., D.P.M. .. .. .. .. .. .. .. .. 116

The Thirty-fourth Long Fox Memorial Lecture: Plant Hormones. By M. Skene, D.Sc. .. .. .. .. .. .. .. .. 125

" Night-Nurse's Paralysis " : A Temporary Tonic Motor Paralysis. By G. de M. Rudolf, M.R.C.P., D.P.M., D.P.H. .. .. .. .. .. 132

Campaigning Notes, 1940–45. By Colonel Martin Herford, D.S.O., M.B.E., M.C., M.B., Ch.B., R.A.M.C. .. .. .. .. .. 136

Reviews of Books .. .. .. .. .. .. .. .. 43, 84, 119, 148

Editorial Notes .. .. .. .. .. .. .. .. .. 47, 150

Obituary .. .. .. .. .. .. .. .. .. .. 152

Meetings of Societies .. .. .. .. .. .. .. .. 48, 121, 153

The Medical Library of the University of Bristol .. .. 50, 86, 157

Publications Received .. .. .. .. .. .. .. .. 54, 87, 160

Local Medical Notes .. .. .. .. .. .. .. 55, 88, 121, 160

### The Medical Library of the University of Bristol

WITH WHICH IS INCORPORATED

**The Library of the Bristol Medico-Chirurgical Society**

---

*The following donations have been received since the publication of the list in the last issue.*

| | |
|---|---|
| Brompton Hospital (1) | 1 Volume. |
| Carlson, Capt. (2) | 4 Volumes. |
| General Medical Council (3) | 1 Volume. |
| Dr. H. B. Howell (4) | 1 Volume. |
| Lefanu, W. R., Esq. (5) | 1 Volume. |
| H. K. Lewis & Co., Ltd. (6) | 1 Volume. |
| Michell Clarke Memorial Fund (7) | 51 Volumes. |
| Royal College of Surgeons (8) | 1 Volume. |
| Surgeon General's Library (9) | 4 Volumes. |
| Trade Delegation of U.S.S.R. (10) | 1 Volume. |
| Watson-Williams, E. (11) | 1 Volume. |
| Wilmer Opthalmological Inst. (12) | 1 Volume. |

Unbound periodicals have also been received from Professor C. Bruce Perry, J. C. Shipham, Esq. and Messrs. John Wright and Sons, Ltd.

### THE ONE HUNDRED AND EIGHTIETH LIST OF BOOKS.

The figures in round brackets refer to the figures after the names of the donors. The books to which no such figures have been attached have either been bought from the Library Fund or received through the *Journal*.

**Appleton, J. L. T.** .. *Bacterial Infections* .. .. .. .. .. 3rd Ed. (7) 1944

**Beaumont, G. E.** .. .. *Medicine* .. .. .. .. .. .. .. 4th Ed. (7) 1945

**Behan, R. J.** .. .. .. *Pain* .. .. .. .. .. .. .. .. .. .. .. (2) 1915

**Blacker, C. P.** .. .. *Neurosis and the Mental Health Services* .. .. 1946

**Blair, V. P., and Ivy, R. H.** *Essentials of Oral Surgery* .. 3rd Ed. (7) 1944

**Bois-Raymond, R.** .. *Physiologie des Menschen und du Sängethiere* (2) 1908

**Bourne, A. W., and Williams, L. H.** *Recent Advances in Obstetrics and Gynæcology* .. .. .. .. .. .. .. .. 6th Ed. 1945

**Boyd, W.** .. .. .. *Pathology of Internal Diseases* .. 4th Ed. (7) 1944

**Boyd, W.** .. .. .. *Surgical Pathology* .. .. .. .. .. 5th Ed. (7) 1945

**Brailsford, J. F.** .. .. *Radiology of Bones and Joints* .. 3rd Ed. (7) 1944

*British Pharmaceutical Codex*, 1934 .. .. .. .. 7th Supplement (7) 1945

**Cameron, A. T.** .. .. *Recent Advances in Endocrinology* 5th Ed. (7) 1945

**Chamberlain, E. Noble** .. *A.B.C. of Medical Treatment* .. .. .. .. .. 1946

**Chesterman, J. T.** .. *Acute Intestinal Obstruction* .. .. .. .. .. (7) 1945

**Clark, W. E. le G.** .. *Tissues of the Body* .. .. .. .. 2nd Ed. (7) 1945

*Cookery Book for Diabetics* (Diabetic Association) .. .. .. .. .. .. .. 1945

**Fifield, L. R.** .. .. *Infections of the Hand* .. .. .. .. .. 2nd Ed. 1939

**Foote, R. R.** .. .. .. *Varicose Veins, Hæmorhoids and other Conditions* .. .. .. .. .. .. .. .. .. .. .. (7) 1944

**Fox, Edw. Long.** .. .. *Cerebral Localisation* (M.S.) .. .. .. .. .. (11)

**French, H.** .. .. .. *Index of Differential Diagnosis* .. .. 6th Ed. 1945

**Gabriel, W. B.** .. .. *Principles and Practice of Rectal Surgery* 3rd Ed. (7) 1945

**Geschickter, C. F.** .. *Diseases of the Breast* .. .. .. 2nd Ed. (7) 1943

**Gillespie, N. A.** .. .. *Endotracheal Anæsthesia* .. .. .. .. .. (7) 1941

**Groves, E. W. Hey** .. *Synopsis of Surgery* .. .. .. 12th Ed. 1945

**Hewer, C. L.** .. .. .. *Recent Advances in Anæsthesia and Analgesia* 5th Ed. (7) 1946

**Howell, H. B.** .. .. *Medicine in Canada* (*Clio Medica*) .. .. (4) 1933

**Human, J. U.** .. .. *Blind Intubation and the Signs of Anæsthesia* 2nd Ed. (7) 1941

**Hunter, D.** .. .. .. *Industrial Toxicology* .. .. .. .. .. .. .. 1944

**Hutchison, Sir R.** .. *Elements of Medical Treatment* .. .. 4th Ed. 1945

**Hutchison, Sir R.** .. *Lectures on Dis. of Children* .. .. 9th Ed. (7) 1945

**Illingworth, C. F. W.** .. *Short Text-book of Surgery* .. .. 3rd Ed. (7) 1943

*Index Catalogue of the Surgeon General's Library*, 4th Series, Vols. 6–9 (9) 1941–45

**Jordan, E. O., and Burrows, W.** *Text-book of Bacteriology* .. 14th Ed. (7) 1945

**Kersley, G. D.** .. .. *Outlines of Physical Methods in Medicine* .. .. 1945

**Kettle, E. H.** .. .. *Pathology of Tumours*, 3rd Ed. by W. G. Barnard and A. H. T. Robb-Smith .. .. .. (7) 1945

| | | | | |
|---|---|---|---|---|
| **Le Fanu, W. R.** | *John Hunter—A List of his Books* | | (5) | 1946 |
| **Lettsom, J. C.** | *History of Some of the Effects of Hard Drinking* | | | 1791 |
| *Lewis's :* 1844–1944 | | | (6) | 1945 |
| **Lewis, Sir T.** | *Exercises in Human Physiology* | | (7) | 1946 |
| **Love, R. J. M.** | *Minor Surgery* | 2nd Ed. | (7) | 1945 |
| **McClance, R. A., and Widdowson, E. M.** | *Chemical Composition of Foods* | 6th Ed. | | 1946 |
| **Mackie, T. J., and McCarling, J. E.** | *Handbook of Practical Bacteriology* | 7th Ed. | (7) | 1943 |
| **McLachlan, A. E. W.** | *Handbook of the Diagnosis and Treatment of Venereal Diseases* | 2nd Ed. | (7) | 1945 |
| **Maxwell, J.** | *Introduction to Diseases of the Chest* | 2nd Ed. | | 1945 |
| **Meakins, J. C.** | *Practice of Medicine* | 4th Ed. | (7) | 1944 |
| **Mennell, J. B.** | *Physical Treatment by Movement and Massage* | 5th Ed. | (7) | 1945 |
| *Microbiology and Epidemiology,* by various authors | | | (10) | 1945 |
| **Minnitt, R. J.** | *Gas and Air Analgesia* | 2nd Ed. | (7) | 1944 |
| **Morley, M. E.** | *Cleft Palate and Speech* | | (7) | 1945 |
| **Myrdal, A.** | *Nation and Family* | | (7) | 1945 |
| *New and Non-Official Remedies* | | | (7) | 1945 |
| **Odgers, P. N. B.** | *Class-Book of Practical Embryology* | | | 1945 |
| **Orban, B.** | *Oral Histology and Embryology* | | (7) | 1944 |
| **Panton, P. N., and Marrack, J. R.** | *Clinical Pathology* | 5th Ed. | (7) | 1945 |
| **Parsons, Sir J. H.** | *Diseases of the Eye* | 10th Ed. | (7) | 1944 |
| **Paterson, D.** | *Sick Children* | 5th Ed. | (7) | 1944 |
| **Paterson, D., and Smith, J. F.** | *Modern Methods of Feeding in Infancy and Childhood* | 8th Ed. | (7) | 1945 |
| **Price, F. W. (Ed.)** | *Practice of Medicine* | 7th Ed. | | 1946 |
| *Queen Charlotte's Text-book of Obstetrics* | | 6th Ed. | (7) | 1945 |
| **Robbins, B. H.** | *Cyclopropane Anæsthesia* | | (7) | 1940 |
| **Rowbotham, G. F.** | *Acute Injuries of the Head* | 2nd Ed. | (7) | 1944 |
| **Rowbotham, S.** | *Anaesthesia in Operations for Goitre* | | (7) | 1945 |
| **Shaw, W.** | *Text-book of Gynæcology* | 4th Ed. | (7) | 1945 |
| **Sheldon, W.** | *Diseases of Infancy and Childhood* | 4th Ed. | (7) | 1945 |
| **Smith, S. and Glaister, J.** | *Recent Advances in Forensic Medicine* | 2nd Ed. | (7) | 1939 |
| **Stevenson, R. Scott** | *Ear, Nose and Throat in the Services* | | | 1943 |
| **Stitt, E. R.** | *Diagnosis, Prevention and Treatment of Tropical Diseases,* 7th Ed. by R. P. Strong | | (7) | 1945 |
| **Stokes, J. H., Burman, H., and Ingraham, N .R.** | *Modern Clinical Syphilology* | 3rd Ed. | (7) | 1945 |
| **Strümpell, A.** | *Lehrbuch du Spiziellen Pathologie und Therapie* 16te Auft, 2 Bde | | (2) | 1907 |
| **Thomas, E. W. Caryl** | *Synopsis of Forensic Medicine and Toxicology* | 2nd Ed. | | 1945 |
| **Topley, W. W. C., and Wilson, G. S.** | *Principles and Practice of Bacteriology and Immunity* | 3rd Ed., 2 vols. | (7) | 1946 |
| **Trail, R. R.** | *Chest Examinations* | 2nd Ed. | (7) | 1945 |
| **Walshe, F. M. R.** | *Diseases of the Nervous System* | 4th Ed. | (7) | 1946 |

**Watson-Jones, Sir R.** .. *Fractures and Joint Injuries* 3rd Ed., 2 vols. (7) 1946

**Whitby, Sir L. E. H., and Britton, C. J. C.** *Disorders of the Blood* 5th Ed. (7) 1946

**White, P. D.** .. .. .. *Heart Disease* .. .. .. .. .. .. 3rd Ed. (7) 1945

**Wright, Samson** .. .. *Applied Physiology* .. .. .. .. .. 8th Ed. 1945

**Zinsser, H. et al** .. .. *Immunity* .. .. .. .. .. .. .. 5th Ed. (7) 1945

TRANSACTIONS, REPORTS, JOURNALS, ETC.

Annual Report of the Smithsonian Institute .. .. .. .. .. 1944 and 1945

Brompton Hospital Reports. Vol. 13 .. .. .. .. .. .. .. .. .. 1944

Collected Reprints : Wilmer Ophthalmological Institute. Vol. 7, 1942–45 (12) 1945

Medical Directory .. .. .. .. .. .. .. .. .. .. .. .. .. .. 1946

Medical Register .. .. .. .. .. .. .. .. .. .. .. .. .. (3) 1946

Proceedings of the University of Otago Medical School .. .. .. .. 1946

Progressive Medicine : March and June, 1924 .. .. .. .. .. .. 1924

Quarterly Cumulative Index Medicus. January to June .. .. .. .. 1945

Royal College of Surgeons of England. Calendar .. .. .. .. .. 1945

Transactions of the Medical Society of London. Vol. 63 .. .. .. 1940–43

Transactions of the Ophthalmological Society, U.K. 64 .. .. .. .. 1944

Yearbook of Radiology .. .. .. .. .. .. .. .. .. .. .. .. 1945

### Publications Received

FROM OXFORD UNIVERSITY PRESS :

*Applied Physiology.* By SAMSON WRIGHT. Eighth Edition. 1945. Price 30s.

*Neurosis and the Mental Health Services.* By C. P. BLACKER. 1946. Price 21s.

*Textbook of the Practice of Medicine.* By various authors. Edited by F. W. PRICE. Seventh Edition. 1946. Price 42s.

*Early Diagnosis of the Acute Abdomen.* By Z. COPE. Ninth Edition. 1946. Price 12s. 6d.

*ABC of Medical Treatment.* By E. N. CHAMBERLAIN. 1946. Price 10s. 6d.

*Cancer of the Scrotum in Relation to Occupation.* By S. A. HENRY. 1946. Price 15s.

From JOHN WRIGHT & SONS LTD. :

*British Journal of Surgery.* Vol. 33, Nos. 131 and 132. January and April, 1946. Price 12s. 6d. per number. Annual subscription, 42s.

*Leprosy.* By Sir LEONARD ROGERS and E. MUIR. Third Edition. 1946. Price 25s.

*The Causation of Appendicitis.* By A. RENDLE SHORT. 1946. Price 10s.

*Medical Aspects of Growing Old.* By A. T. TODD. 1946. Price 15s.

From H. K. LEWIS & CO. LTD. :

*Cardiovascular Disease in General Practice.* By T. EAST. Second Edition. 1946. Price 12s. 6d.

*Theory and Practice of Nursing.* By M. A. GULLAN. 1946. Price 12s. 6d.

From HENRY SCHUMAN, NEW YORK :

*Journal of the History of Medicine.* Vol. 1, No. 1. 1946. Price $2.50 per number. Annual subscription, $8.50.

### Local Medical Notes

THE Medical Library of the University is now open, for the convenience of members, until 7 p.m. on Mondays and Fridays.

The next Long Fox Memorial Lecture will be delivered, probably in November, by Dr. Kenneth S. Bergin, Assistant Director of Medical Services, B.O.A.C., on " Some Aspects of Aviation Medicine."

BRISTOL UNIVERSITY.

The following Appointments of Lecturers have been made: S. T. Crowther, M.R.C.S., L.R.C.P., D.P.H., *in Bacteriology;* M. P. Embrey, M.D., B.Sc., F.R.C.S., M.R.C.O.G., *in Obstetrics;* W. A. Gillespie, M.A., M.D., M.R.C.P.I., D.P.H., *in Clinical Pathology;* W. Hobson, M.D., B.Sc., D.P.M., *in Preventive Medicine;* G. K. McGowan, B.M., B.Ch., *in Clinical Pathology;* G. A. Smart, M.D., B.Sc., M.R.C.P., *in Medicine;* Capt. John Wainwright, M.B., Ch.B., *in Pathology;* E. Watson-Williams, Ch.M., F.R.C.S., *in Laryngology, Rhinology and Otology.* G. H. Tovey, M.D., *Special Lecturer in Hœmatology.*

EXAMINATION RESULTS.

Students of the University have passed the following examinations:—

**University of Bristol.**—M.B., CH.B.—*Second Examination (Section I):* Rosalind P. Adams, Joan Barnes, B. B. Beeson, D. J. Brain, Joan K. Cheeseman, Jennifer M. Comely (*a*), A. R. Ebanks, C. C. Ewing, P. C. Farrant, Katharine E. Fawcus, Elizabeth M. Fraser, D. M. Gambier, Patricia M. Gilbert, R. B. Handscombe, Pamela Hawkins, Pamela M. Hellings, Sybil E. Jacob, Ellen M. Marron, J. P. Martin, Elizabeth M. Morgan, P. A. Normandale, Margaret R. Salisbury, H. Schnieden, J. D. Sheward, June M. Smith, Medora Storkey, G. Walters, Olwen J. West (*b*), Catherine C. White.

(*a*) With distinction in Anatomy.
(*b*) With distinction in Physiology.

**Royal College of Surgeons.**—F.R.C.S.—*Primary Examination:* C. H. Bartlett, Marjorie O. Dunster, A. G. H. Mitchinson, A. N. H. Peach.

**Conjoint Board.**—M.R.C.S., L.R.C.P.—Pamela M. Appleton (*e*), J. H. Beatson (*c, d*), W. H. K. Carpenter (*c, d, f*), P. J. Chapman (*c, d*), Pamela M. Farmer (*f*), N. G. Glen (*c*), Mrs. E. W. Higgens (*c, d*), F. D. Kay (*c*), M. B. Lennard (*c, g*), R. G. G. Taylor (*d*), W. Wagland (*d, g*).

(*c*) Midwifery.
(*d*) Pathology.
(*e*) Pharmacology.
(*f*) Medicine.
(*g*) Surgery.

APPOINTMENTS.

BRISTOL ROYAL HOSPITAL HONORARY MEDICAL STAFF:

Dr. H. H. Carleton, M.A., D.M., B.Ch., F.R.C.P., and Dr. C. E. K. Herapath, M.C., M.D., B.S., have been appointed Honorary Consulting Physicians.

Professor Rendle Short, M.D., B.S., B.Sc., F.R.C.S., and Mr. H. Chitty, M.S., F.R.C.S., have been appointed Honorary Consulting Surgeons.

Mr. A. J. Wright, M.B., B.S., B.A., F.R.C.S., has been appointed Honorary Consulting Surgeon, and Mr. G. R. Scarff, M.B., Ch.B., F.R.C.S., and Mr. J. Angell James, M.D., M.R.C.P., F.R.C.S., Honorary Surgeons to the Ear, Nose and Throat Department.

Mr. F. J. Hector, M.D., Ch.B., M.R.C.O.G., has been appointed Honorary Obstetrician.

Mr. K. H. Pridie, M.B., Ch.B., F.R.C.S., has been appointed Honorary Surgeon to the Fracture and Orthopædic Department.

### Bristol Medico-Chirurgical Society

### President.

H. H. CARLETON, M.A., M.D. Oxon., F.R.C.P.

### President-Elect.

H. CHITTY, M.S. Lond., F.R.C.S.

### Committee.

*Ex-Officio* { J. A. NIXON, *C.M.G.*, B.A., M.D. Cantab. (Editor of *Journal*).

(Vacant), (Hon. Librarian).

*Elected.*

C. CORFIELD, M.D. (Ex-President).
S. BRYAN ADAMS, Ph.D, M.B., Ch.B.
R. F. BARBOUR, M.A., F.R.C.P., D.P.M.
F. H. BODMAN, M.D.
BERYL D. CORNER, M.D.
T. F. HEWER, M.D.
G. D. KERSLEY, M.A., M.D., F.R.C.P.
G. L. ORMEROD, M.A., M.B., B.Chir.
H. K. V. SOLTAU, M.D.

### Honorary Secretary.

A. L. EYRE-BROOK, M.S.

### Honorary Treasurer.

FREDERICK SUTTON, M.C., M.D.

### University Library Sub-Committee.

A. R. SHORT, B.Sc., M.D. Lond., F.R.C.S.
C. BRUCE PERRY, M.D. Brist., F.R.C.P.
H. J. DREW SMYTHE, *M.C.*, M.S., M.D. Lond., F.R.C.S., F.R.C.O.G.

Medical Practitioners wishing to join the Society are requested to communicate with the Hon. Secretary, Mr. A. L. Eyre-Brook, Litfield House, Clifton Down, Bristol 8, The Annual Subscription is £1 1s., payable to Hon. Treasurer.

---

*Extracts from Journal By-laws.*

4.—The *Journal* shall be delivered free to Subscribers and also to Members of the Society whose subscriptions are not in arrear.

6.—The Annual Subscription to the *Journal* is 10/6, payable to Hon. Treasurer.

---

Communications intended for insertion in the *Journal* should be sent to the Editor, Dr. J. A. Nixon, 7 Lansdown Place, Clifton, Bristol.

Books for Review and Exchange Journals should be sent to the Assistant Editor, *Bristol Medico-Chirurgical Journal*, Medical Library, The University, Bristol. Reviews should be returned with the books to Mr. W. M. Capper, Litfield House, Clifton Down, Bristol 8.

Communications referring to the delivery of the *Journal* should be sent to the Hon. Treasurer, Dr. Frederick Sutton, 1 Pembroke Road, Bristol 8.

Volumes of the *Journal* can be bound upon application to J. W. Arrowsmith Ltd., Quay Street, Bristol, price on request. The inclusive Index for Volumes I—LI may be obtained, price 3/– (postage extra).

### Bristol Medico-Chirurgical Society

### PAST PRESIDENTS

1874—75. FREDERICK BRITTAN, M.D., B.S. Dub.
1875—76. ROBERT WILLIAM COE.
1876—77. SAMUEL MARTYN, M.D. Ed.
1877—78. AUGUSTIN PRICHARD, M.D. Berlin.
1878—79. HENRY EDWARD FRIPP, M.D. Würzburg.
1879—80. WILLIAM MICHELL CLARKE.
1880—81. JOSEPH GRIFFITHS SWAYNE, M.D. Lond.
1881—82. EDWARD LONG FOX, M.D. Oxon.
1882—83. JAMES GEORGE DAVEY, M.D. St. And.
1883—84. WILLIAM JOHNSTONE FYFFE, M.D., B.A. Dub.
1884—85. GEORGE FORSTER BURDER, M.D. Aberd.
1885—86. WILLIAM HENRY SPENCER, M.D., M.A. Cantab.
1886—87. FRANCIS POOLE LANSDOWN.
1887—88. LEMUEL MATTHEWS GRIFFITHS.
1888—89. ROBERT SHINGLETON SMITH, M.D., B.Sc. Lond.
1889—90. NELSON CONGREVE DOBSON, Ch.M. Brist.
1890—91. SAMUEL HENRY SWAYNE.
1891—92. FRANCIS RICHARDSON CROSS, M.B. Lond., LL.D. Brist.
1892—93. EDWARD MARKHAM SKERRITT, M.D., B.S., B.A. Lond.
1893—94. JAMES GREIG SMITH, M.B., C.M., M.A. Aberd., F.R.S.E.
1894—95. ALFRED JAMES HARRISON, M.B. Lond.
1895—96. ARTHUR WILLIAM PRICHARD.
1896—97. ALFRED EDWARD AUST LAWRENCE, M.D., C.M. Aberd.
1897—98. JOHN EDWARD SHAW, M.B., C.M. Ed.
1898—99. ROBERT ROXBURGH, M.B. Ed.
1899—00. WILLIAM HENRY HARSANT.
1900—01. DAVID SAMUEL DAVIES, M.D. Lond.
1901—02. BARCLAY JOSIAH BARON, M.B., C.M. Ed.
1902—03. GEORGE MUNRO SMITH, M.D. Brist.
1903—04. JAMES PAUL BUSH, C.M.G., C.B.E., Ch.M. Brist
1904—05. REGINALD EAGER, M.D. Lond.
1905—06. JOHN DACRE.
1906—07. JAMES TAYLOR.
1907—08. HENRY WALDO, M.D., C.M. Aberd.
1908—09. JOHN MICHELL CLARKE, M.D., M.A. Cantab.
1909—10. JAMES SWAIN, C.B., C.B.E., M.D., M.S. Lond.
1910—11. BERTRAM MITFORD HERON ROGERS, M.D., B.Ch., B.A. Oxon.
1911—12. CHARLES ALEXANDER MORTON.
1912—13. WALTER CARLESS SWAYNE, M.D., B.S. Lond. and Brist.
1913—14. PATRICK WATSON-WILLIAMS, M.D. Lond., M.D. (Hon. Causa) Brist.
1914—15. WILLIAM HARRY CHRISTOPHER NEWNHAM, M.A., M.B. Cantab.
1915—16. GEORGE PARKER, M.A., M.D. Cantab., LL.D. Brist.
1916—17. FRANCIS HENRY EDGEWORTH, M.A., M.D. Cantab., D.Sc. Lond.
1917—18. FREDERICK LACE.
1918—19. ROBERT GUTHRIE POOLE LANSDOWN, M.D., B.S. Durh.
1919—20. I. WALKER HALL, M.D., Ch.B. Vict.
1920—21. LEOPOLD ERNEST VALENTINE EVERY-CLAYTON, M.D. Lond.
1921—22. CYRIL HUTCHINSON WALKER, B.A., M.B. Cantab.
1922—23. JOHN ALEXANDER NIXON, C.M.G., B.A., M.D. Cantab.
1923—24. JOHN LACY FIRTH, M.D., M.S. Lond.
1924—25. JOHN ODERY SYMES, M.D. Lond.
1925—26. THOMAS CARWARDINE, M.S. Lond.
1926—27. ARTHUR LAUNCELOT FLEMMING, M.B., Ch.B. Brist.
1927—28. WALTER KENNETH WILLS, M.A., M.B., B.Ch. Cantab.
1928—29. HENRY LAWRENCE ORMEROD, M.D., B.Ch. R.U.I.
1929—30. CHARLES FERRIER WALTERS.
1930—31. DAVID CHARLES RAYNER, Ch.M. Brist.
1931—32. JOHN ROGER CHARLES, M.A., M.D. Cantab.
1932—33. ERNEST WILLIAM HEY GROVES, M.D., M.S., D.Sc., B.Sc. Lond.
1933—34. HERBERT ELWIN HARRIS, B.A., M.B. Cantab.
1934—35. A. RENDLE SHORT, B.Sc., M.D. Lond.
1935—36. ARTHUR ERNEST ILES, O.B.E., M.B., B.S. Lond.
1936—37. RICHARD CHRISTOPHER CLARKE, O.B.E, M.B. Lond.
1937—38. CLIFFORD A. MOORE, M.B., M.S. Lond.
1938—40. LEONARD AUGUSTUS MOORE, M.B., Ch.B. Brist.
1940—41. CHARLES CORFIELD, M.D. Durh., Barr.-at-Law.
1945—46. H. H. CARLETON, M.A., M.D. Oxon., F.R.C.P. Lond.

### LIST OF SUBSCRIBERS

TO THE

### Bristol Medico-Chirurgical Journal.

### HONORARY MEMBER OF THE SOCIETY.

SWAIN, JAMES, *C.B.*, *C.B.E.*, M.D., M.S. Lond. .. .. .. Wyndham House, Easton-in-Gordano.

### SUBSCRIBERS.

*Those marked * are Members of the Society.*

| | |
|---|---|
| *ADAMS, A. W., M.S. Lond. .. .. .. .. | Elton House, Clifton, Bristol 8. |
| ADAMS, J. B. .. .. .. .. .. .. .. .. | Kent Lodge, Eastbourne. |
| *ADAMS, S. B., Ph.D., M.B., Ch.B. Brist. .. | Clapton Hill, near Portishead. |
| *ALDWINCKLE, HELEN M., M.B., Ch.B. Brist... | 23 Rockland Road, Downend, Bristol. |
| *ALEXANDER, A. J. P., M.D. Belf. .. .. .. | Winsley Sanatorium, near Bath. |
| *ALEXANDER, D. A., M.B., Ch.B. Vict. .. .. | 112 Pembroke Road, Clifton, Bristol 8. |
| ALEXANDER, G. L., B.A., M.B., B.Ch. Cantab. | West African Medical Service, Laura, via Accra, Gold Coast. |
| *ALFORD, A. F., M.B., Ch.B. Brist. .. .. | Ministry of Education, Belgrave Square, London, S.W.1. |
| ALFORD, H. J., M.D. Lond. .. .. .. .. .. | 4 Park Terrace, Taunton. |
| ASHTON, L. P., M.B., Ch.B. Brist. .. .. .. | Kapsowar, P.O. Eldoret, Kenya, East Africa. |
| *BAER, P., M.D. .. .. .. .. .. .. .. .. | 10 Redland Park, Bristol 6. |
| *BAIN, W. .. .. .. .. .. .. .. .. .. | 1 Greville Road, Southville, Bristol 3. |
| *BAKER, LILY A., M.D., B.Ch., B.A.O., R.U.I. | 23 Pembroke Road, Clifton, Bristol 8. |
| BALL, Mrs. J. J. .. .. .. .. .. .. .. | St. Leonard's Vicarage, Redfield, Bristol 5. |
| *BARBOUR, R. F., .. .. .. .. .. .. .. | 13 Old Sneed Road, Stoke Bishop, Bristol. |
| *BAXTER, URSULA, M.B., Ch.B. Brist. .. .. | 167 Wells Road, Knowle, Bristol 4. |
| *BAXTER, V. T., M.B., Ch.B. .. .. .. .. | 167 Wells Road, Knowle, Bristol 4. |
| *BEATSON, D. H., M.B., Ch.B. Brist. .. .. | 8 Richmond Park Road, Clifton, Bristol 8. |
| *BECK, DIANA J. KINLOCH, M.B., B.S. .. .. | Newlands, Frenchay. |
| *BELL, R. J. IRVING .. .. .. .. .. .. | 5a Oakfield Road, Clifton, Bristol 8. |
| *BERGIN, F. G. .. .. .. .. .. .. .. .. | 31 Oakfield Road, Clifton, Bristol 8. |
| *BERRY, R. J. A., Prof., M.D., C.M. Edin. .. | 30 All Saints' Road, Clifton, Bristol 8. |
| *BIRRELL, J. A., M.D., B.S. Lond. .. .. .. | 1 Victoria Square, Bristol 8. |
| *BISHOP, G. W. R., O.B.E., M.B., Ch.B. Brist. | Bloomfield House, High Street, Shepton Mallet. |
| BLACKETT, J. F., M.D., B.S. Lond... .. .. | Hamsterley, Bloomfield Park, Bath. |
| BLETCHLY, G. PLAYNE, M.B. Lond. .. .. | c/o Lloyds Bank, Nailsworth, Glos. |
| *BODMAN, F. H., M.D., Ch.B. Brist... .. .. | 2 Redland Court Road, Bristol 6. |
| BOLT, R. F. .. .. .. .. .. .. .. .. .. | 70 Great Russell Street, London, W.C.1. |
| *BOULTON, B. J., M.B., Ch.B. Brist. .. .. | Ham Green Hospital, Pill, near Bristol. |
| *BOWN, NAOMI J., M.B., Ch.B. Brist. .. .. | The Corner, Nailsea, Somerset. |
| *BOYD, R. G., M.B., Ch.B. .. .. .. .. .. | Bristol General Hospital. |
| *BRADBEER, E. G., M.B., Ch.B. Brist. .. .. | 13 Percival Road, Bristol 8. |
| BRASHER, C. W. J., M.D. .. .. .. .. .. | Murivance, Nether Stowey, Somerset. |
| *BRINCKMAN, WINIFRED P., M.B., Ch.B. Brist. | 2 Clifton Park, Bristol 8. |
| *BRITTON, R. B., M.B., B.S. Lond. .. .. .. | 33a Whiteladies Road, Clifton, Bristol 8. |
| *BROCKLEHURST, R. J., M.A., D.M. Oxon. .. | The University, Bristol 8. |
| BROWN, J. E., M.B., Ch.B. Brist. .. .. .. | Coulthurst Villa, Brives Road, Maraval, Trinidad, B.W.I. |
| BRYAN, C. .. .. .. .. .. .. .. .. .. | 44 Nevil Road, Bishopston, Bristol 7. |
| *BRYANT, EUNICE .. .. .. .. .. .. .. | 167 Redland Road, Bristol 6. |
| *BURGES, R. .. .. .. .. .. .. .. .. .. | Druid's Mead, Stoke Bishop, Bristol 9. |
| *BUSH, G. B., M.B., Ch.B. Brist. .. .. .. | Litfield House, Clifton, Bristol 8. |

| | |
|---|---|
| *CAIRNS, F. J. | Devon House, Whitehall Road, Bristol 5. |
| CAMERON, MARGARET, M.B., Ch.B. | 1 Dean Lane, Bristol 3. |
| *CAPPER, W. M., M.B., B.S. Lond. | Litfield House, Clifton, Bristol. |
| *CAREY, R. S., *O.B.E.*, B.A. Cantab. | 502 Bath Road, Brislington, Bristol 4. |
| *CARLETON, H. H., M.A., M.D., B.Ch. Oxon. | 1 Lansdown Road, Clifton, Bristol 8. |
| *CASSON, E., M.D., Ch.B. Brist. | Mount Pleasant, Clevedon. |
| *CHAMBERS, E. R. | 9 Clifton Park, Clifton, Bristol 8. |
| *CHARLES, J. R., M.A., M.D. Cantab. | Litfield House, Clifton Down, Bristol 8. |
| *CHITTY, H., M.S. Lond. | Naish House, Clapton-in-Gordano. |
| *CHRISTOFFERSON, P. E., M.B., Ch.B. Brist. | 14 Oakfield Road, Clifton, Bristol 8. |
| *CLAREMONT, L. E., M.D.S. Brist. | 5 Rodney Place, Clifton, Bristol 8. |
| *CLARK, DENNIS O. | 168 Milton Road, Weston-super-Mare. |
| *CLARKE, R. C., *O.B.E.*, M.B., Ch.B. Brist. | Harley Lodge, Clifton Park, Bristol 8. |
| *COATES, H. | Notts County Mental Hospital, Radcliffe-on-Trent. |
| *COCHRANE, J. H. | 11 Bryant's Hill, St. George, Bristol 5. |
| *COLLINGWOOD, F. C., M.B., Ch.B. Brist. | 106 Foxley Lane, Purley, Surrey. |
| *COOKE, ELIAZBETH M., M.D. Lond., M.R.C.P. | 53 Queen's Court, Clifton 8. |
| *COOKE, R. V., Ch.M. Brist., F.R.C.S. | Litfield House, Bristol 8. |
| *COOKSON, R. G. | Clifton College, Bristol 8. |
| COOMBS, N. C. | Romaldkirk, Barnard Castle, Co. Durham. |
| *COOPER, W. R. | 13 Westfield Park, Redland, Bristol 6. |
| *CORFIELD, C., M.D. Durh. | 5 Kensington Place, Clifton, Bristol 8. |
| *CORNER, BERYL, M.D., B.S. Lond. | 3 Elton House, Rodney Place, Bristol 8. |
| *COSSHAM, W. L., M.B., Ch.B. Brist. | 3 Claremont Road, Bishopston, Bristol 7. |
| *COURTNEY, R. M., M.B., Ch.B. Glasg. | Manor House, Southmead Road, Bristol |
| *COXON, BEATRICE | 16 Richmond Hill, Clifton, Bristol 8. |
| *CRAIG, ANNE A., M.D., B.S. Lond. | 35 Queen's Court, Clifton, Bristol 8. |
| *CROOK, B. A., M.B., Ch.B. Brist. | The Laurels, Timsbury, near Bath. |
| CROOT, Lt.-Col. H. J., R.A.M.C. | Indian Gen. Hosp. (C), India Command. |
| *CROSSMAN, F. W., M.B. Durh. | White's Hill, Hambrook, near Bristol. |
| | |
| *DATTA, S. S., M.D. Brist. | Snowdon House, Fishponds, Bristol. |
| *DAVIES, E. D. D. | Standish House, Stonehouse, Glos |
| *DAVIES, J. N. P., M.B., Ch.B. | c/o. Dir. Med. Services, Entebbe, Uganda. |
| *DELANEY, N., M.B., Ch.B. Manch. | The Meade, Bishopsworth, Somerset. |
| *DISNEY, M., M.D. Lond. | 2 Clifton Park, Bristol 8. |
| *DIX, C. | 14 Mortimer Road, Clifton, Bristol 8 |
| *DIXON, MARY, M.B., Ch.B. | Bristol Maternity Hospital. |
| *DIXON, T. B., C.B.E., V.D. | 11 Pembroke Road, Bristol 8. |
| *DODD, GRANTHAM, M.B., B.S. | 42 Woodstock Road, Bristol 6. |
| *DOWLING, A.A., M.B., Ch.B. Brist. | 13 Dowry Square, Bristol 8. |
| *DUERDEN, J. R., M.B., Ch.B. Brist. | 4 Cecil Road, Bristol 8. |
| *DUNSTER, MARJORIE, M.D., Ch.M. | Bristol General Hospital (St. Monica's). |
| *DUTTON, HILDA R. | 405 Stapleton Road, Bristol 5. |
| | |
| *EGLINTON, J. H. C. | Street, Somerset. |
| ELLIOTT, F. P., M.B., C.M. Aberd. | 113 Grove Road, Walthamstow, London, E. 17. |
| *ELLIOTT, W. H. A., M.B., B.S. Lond. | 29 Old Sneed Avenue, Stoke Bishop, Bristol 9. |
| *ELLIOTT, LIEUT.-COMM. JOHN, M.B., Ch.B. | 29 Old Sneed Avenue, Bristol 9. |
| *ELLIOTT, S.Q-LEADER W. H. GREVILLE, M.B., Ch.B. | 29 Old Sneed Avenue, Bristol 9. |
| *EMERY, J. L. | Childrens' Hospital, Bristol 2. |
| *EVANS, G. M., M.B., Ch.B. Brist. | 117 Ashley Road, Bristol. |
| *EYRE-BROOK, A. L., M.B., M.S. Lond. | Litfield House, Bristol 8. |
| | |
| *FELLS, G. R., M.B., Ch.B. Brist. | 19 Southmead Road, Bristol. |
| *FELLS, R. R., M.B., B.S. Lond. | 17 Mortimer Road, Clifton, Bristol 8. |
| *FENELEY, G. L., M.B., Ch.B. Brist. | Englewood, Falcondale Road, Westbury-on-Trym. |

| | |
|---|---|
| *FISHER, A. C., M.D. Brist. .. .. .. .. | Roan Antelope Mine, N. Rhodesia. |
| *FITZGIBBON, G. M., M.D. Lond. .. .. .. | 1 Stoke Road, Bristol 9. |
| *FORRESTER-BROWN, MAUD, M.D., M.S. Lond. | 22 Combe Park, Bath. |
| *FOSS, G. L., M.D., B.Ch. Cantab. .. .. .. | 1 Cambridge Park, Bristol 6. |
| *FOSS, MISS JOAN, M.B., Ch.B. .. .. .. .. | Clouds Hill House, St. George, Bristol 5. |
| *FOX, F. E., B.A. Cantab. .. .. .. .. .. | Brislington House, Brislington, Bristol 4. |
| *FRASER, A. D., M.A., M.B., Ch.B. Glasg. .. | 4 Alexandra Road, Clifton, Bristol 8. |
| *FRASER, D., M.B., Ch.B. Edin. .. .. .. | Westgate, Dursley, Glos. |
| *FULLER, H. L. .. .. .. .. .. .. .. .. .. | 9 Gay Street, Bath. |
| *FINZEL, H. F. M. .. .. .. .. .. .. .. | The Beeches, St. Anne's Park, Bristol 4. |
| | |
| *GARDEN, R. R., M.A., M.B., B.Ch. Aberd. .. | 9 Harley Place, Clifton, Bristol 8. |
| GARLAND, E. B., M.B., C.M. Edin. .. .. .. | 114a Hampton Road, Redland, Bristol 6. |
| *GERRISH, NORMAN, M.B., Ch.B. Brist. .. .. | 2 Station Road, Keynsham, Som. |
| *GIBBS, J. R. .. .. .. .. .. .. .. .. .. | 48 Codrington Road, Bishopston, Bristol 7. |
| *GOLDING, H. M., *D.F.C.*, M.B., Ch.B. .. .. | 10 Barton Hill Road, Bristol 5. |
| *GORHAM, A. P., M.B., Ch.B. Brist. .. .. .. | 53 Westbury Road, Westbury-on-Trym. |
| *GORNALL, W. A., M.D. Brist. .. .. .. .. | Ulvik, Nailsea, near Bristol. |
| GRIFFITHS, CORNELIUS A. .. .. .. .. .. | 35 Newport Road, Cardiff. |
| | |
| *HAM, C. I., M.B., Ch.B.Brist. .. .. .. .. | 89 Stonebridge Park, Eastville, Bristol. |
| *HARRIS, H. E., M.A., M.B., B.Ch. Cantab. .. | 13 Lansdown Place, Clifton, Bristol 8. |
| *HARTLEY, GRETA, M.D. .. .. .. .. .. .. | 49 Queen's Court, Clifton, Bristol 8. |
| *HAWKINS, MONICA, M.B., Ch.B. .. .. .. | Cowslip Green, Wrington. |
| *HEALES, J. N., M.B., Ch.B. Brist. .. .. .. | Holmcroft, Street, Somerset. |
| *HECTOR, F., M.D. Lond. .. .. .. .. .. .. | 1 Victoria Square, Clifton, Bristol 8. |
| *HEMPHILL, R., M.D. Dub. .. .. .. .. .. | Clonora, Blackberry Hill, Fishponds. |
| *HENDERSON, JAMES, M.B., Ch.B. Aberd. .. | The City Sanatorium, Yardley Road, Birmingham. |
| *HERAPATH, C. E. K., *M.C.*, M.D. Lond. .. | Park Close, 92 Charlton Road, Keynsham. |
| *HEWER, Professor T. F., M.D. Brist. .. .. | Canynge Hall, Bristol 8. |
| *HILL, WINIFRED, M.B., Ch.B. Brist. .. .. | c/o Lloyd's Bank, Ilford. |
| *HILL, L. C., M.D. .. .. .. .. .. .. .. | 11 The Circus, Bath. |
| *HOFFMAN, H. LOVELL, B.A., M.D., B.Ch. Cantab. .. .. .. .. .. .. .. .. | 26 The Circus, Bath. |
| *HOLLAND, W. A. L. .. .. .. .. .. .. .. | 1 Upper Belgrave Road. Clifton. |
| *HOLLAND, L. C. .. .. .. .. .. .. .. .. | Upavon, Wilts. |
| *HOLMES, H. W. H., B.A., M.B. .. .. .. | Burnham-on-Sea, Som. |
| *HOOKER, J. A., Colonel, M.B., Ch.B.Brist. .. | 16 St. Edyth's Road, Sea Mills 9. |
| *HOSKEN, J. G. F... .. .. .. .. .. .. .. | Uley, Glos. |
| HOSPITAL, BRISTOL GENERAL .. .. .. .. .. | |
| *HOUGHTON, LYDIA M., M.B., B.S. Lond. .. | 74 Church Road, Redfield, Bristol 5. |
| *HOWELL, T. E. .. .. .. .. .. .. .. .. | 6 Richmond Hill, Bristol 8. |
| *HUDSON, T. G. FAULKNER, M.D. .. .. .. | Imperial Smelting Co., Avonmouth. |
| *HUGHES, F. W. TERRELL, M.B., Ch.B. Brist. | Chew Magna, Somerset. |
| *HUNTER, F. S. .. .. .. .. .. .. .. .. | Penrose Cottage, Clifton Down, Bristol 8. |
| *HYATT, ANNIE W., M.B., B.S. Lond. .. .. | Merrymead, Shepton Mallet, Som. |
| | |
| *ILES, ANNA, M.B., Ch.B. Brist. .. .. .. | 87 Pembroke Road, Clifton, Bristol 8. |
| *ILES, A. E., *O.B.E.*, M.B., B.S. Lond. .. .. | 87 Pembroke Road, Clifton, Bristol 8. |
| INFIRMARY, BRISTOL ROYAL .. .. .. .. .. | |
| INGLE, C. DURBAN .. .. .. .. .. .. .. | Somerton, Somerset. |
| *ISSERLIN, B. .. .. .. .. .. .. .. .. .. | 21 St. John's Road, Bristol 8. |
| | |
| *JACKMAN, W. A., *T.D.*, M.B., Ch.B. Brist. .. | 15 Mortimer Road, Clifton, Bristol 8. |
| *JACOBSON, L. C., M.B., B.Ch. .. .. .. .. | Bristol General Hospital. |
| *JAMES, J. A., M.D. Lond. .. .. .. .. .. | Litfield House, Clifton Down. Bristol 8. |
| *JAMES, J. A., Senr. .. .. .. .. .. .. .. | Dusk, Elmlea Avenue, Stoke Bishop, Bristol, 9. |
| *JENKINS, F. G., M.B., Ch.B. Brist. .. .. .. | 51 Redcliff Hill, Bristol 1. |
| *JENRINS, R. D. .. .. .. .. .. .. .. .. | The Copper Beeches, Almondsbury, near Bristol. |

| | |
|---|---|
| *JENKINS, P. K., *M.C.*, M.B., Ch.B. .. .. .. | St. Helena, Marine Parade, Clevedon. |
| *JOHNSON, R. G., M.B. Lond. .. .. .. .. | 119 Redland Road, Bristol 6. |
| *JOHNSON-MASON, J. .. .. .. .. .. .. .. | Brentry House, Brentry. |
| *JONES, MEGAN E. .. .. .. .. .. .. .. | Royal Infirmary, Bristol 2. |
| | |
| *KEMM, N. F., M.B., B.S. Lond. .. .. .. .. | 9 Miles Road, Clifton, Bristol 8. |
| *KERSLEY, G. D., M.D. .. .. .. .. .. .. | 6 The Circus, Bath. |
| *KINDERSLEY, C. E., M.B., Ch.B., B.A. Cantab. | 18 The Circus, Bath. |
| KING, E. F. .. .. .. .. .. .. .. .. .. .. | 2 Upper Wimpole Street, London, W.1. |
| *KINGSTON, S. H., M.B., Ch.B. Brist. .. .. | 3 Whiteladies Road, Clifton, Bristol 8. |
| *KYLE, H. G., M.A., M.D., B.Ch. Oxon. .. .. | 31 Westbury Road, Bristol. |
| | |
| *LACE, F .. .. .. .. .. .. .. .. .. .. | 5 Gay Street, Bath. |
| LALONDE, T. P., M.B., Ch.B. Brist... .. .. | Wykeham House, Romsey, Hants. |
| *LEE-MICHELL, R. .. .. .. .. .. .. .. .. | Clifton Lodge, Cheddon Road, Taunton. |
| *LEES, E. LEONARD, M.D., C.M. Edin. .. .. | 28 Downs Park West, Bristol 6. |
| *LEWIS, F. J., M.B., Ch.B. Brist. .. .. .. | Canynge Hall, Bristol 8. |
| *LOGAN, H. B. .. .. .. .. .. .. .. .. | Elmwood, Aberdeen Road, Cotham, Bristol 6. |
| *LOXTON, S. D., M.B., Ch.B. .. .. .. .. | Bristol Royal Infirmary. |
| *LUCAS, J. E. .. .. .. .. .. .. .. .. .. | c/o 132 York Road, Bristol 3. |
| *LUCAS, J. J. S., M.D. Lond. .. .. .. .. | 186 Wells Road, Knowle, Bristol 4. |
| *LUCAS, J. V. .. .. .. .. .. .. .. .. .. | 186 Wells Road, Knowle, Bristol 4. |
| *LUCAS, R. P., M.B., Ch.B. Brist. .. .. .. | Northways, Fallodon Way, Henleaze. |
| | |
| *McKENZIE, W. G., *M.C.* .. .. .. .. .. .. | 11 Bath Road, Reading. |
| *MACLACHLAN, A. M., M.B., Ch.B. Glasg. .. | 65 Redcliff Hill, Bristol 1. |
| *MARSHALL, A. H., M.B., Ch.B. Brist. .. .. | 281 Canford Lane, Bristol. |
| *MARWOOD, S. F., M.D., B.S. .. .. .. .. | Troy House, Kingswood, Bristol. |
| *MACLAREN, CATHERINE J., M.B., Ch.B. Edin. | Hereford House, Clifton Down. |
| MACLEAN, SIR EWEN J., M.D., C.M. Edin. .. | 12 Park Place, Cardiff |
| *MACLEOD, G., M.A. Glas., M.D., M.B. .. .. | Wargrave, Clevedon, Somerset. |
| MARSHALL, A. L., M.B., B.A. Cantab. .. .. | Laxfield, Woodbridge, Suffolk. |
| *MARTIN, P. S., *M.C.* .. .. .. .. .. .. .. | Victoria Quadrant, Weston-super-Mare. |
| *MATHESON, N. R., M.B., Ch.B... .. .. .. | 37 Abbey Road, Westbury-on-Trym. |
| *MAWSON, EVELYN, M.B., Ch.B. .. .. .. | Frenchay Park Sanatorium, Bristol. |
| MIALL, C. L'O .. .. .. .. .. .. .. .. | Elm Hayes, Paulton, near Bristol. |
| *MILLING, T., M.B., Ch.B. Belf. .. .. .. .. | 1 Vicarage Road, Southville, Bristol 3. |
| *MOHAN, S. L., M.B., B.S... .. .. .. .. | 225 Stapleton Road, Bristol 5. |
| *MOORE, C. A., M.B., M.S. Lond. .. .. .. | South Lodge, Kingsweston Road, Henbury, Bristol. |
| *MOORE, L. A., M.B., Ch.B. Brist. .. .. .. | Hillsborough, Cotham Park, Bristol 6. |
| *MOORE, R. H., M.B., Ch.B. Brist. .. .. .. | White Cross Court, Wells Road, Bristol 4. |
| *MUNDY, G. S., M.B., B.Ch. Brist. .. .. .. | The Knoll, Chipping Sodbury, Glos. |
| *MURPHY, F. D., M.B. .. .. .. .. .. .. | 20 Gay Street, Bath. |
| *MUSSEN, R. W., M.D. Belfast, .. .. .. .. | Royal Naval Medical School, Clevedon. |
| *MYLES, Q. St. L., M.A., B.M., B.Ch. Oxon. .. | 7 Apsley Road, Clifton, Bristol 8. |
| | |
| *NICHOLSON-LAILEY, J. R. .. .. .. .. .. | Elmfield House, South Road, Taunton. |
| *NICHOLSON, M. A., M.B., Ch.B. .. .. .. | 2 Elmlea Avenue, Stoke Bishop, Bristol. |
| *NIXON, DOREEN, M.B., B.S. Lond. .. .. .. | 7 Lansdown Place, Clifton, Bristol 8. |
| *NIXON, J. A., *C.M.G.*, M.D. Cantab. .. .. | 7 Lansdown Place, Clifton, Bristol 8. |
| *NOBLE, J. I., M.B., Ch.B. Liverp. .. .. .. | 102 Pembroke Road, Clifton, Bristol 8. |
| *NORMAN, R. M., M.D. Brist. .. .. .. .. | Highwood, Stapleton Park, Bristol. |
| | |
| *O'DONOHOE, MONICA A., M.B., Ch.B. .. .. | 1 Beaconsfield Road, Bristol 8. |
| *ORMEROD, G. L., M.A., M.B., B.Ch. Cantab .. | 2 Henleaze Road, Westbury-on-Trym, Bristol. |
| *ORR-EWING, H. J., *M.C.*, M.D. Lond. .. .. | 1 Beaufort Buildings, Clifton, Bristol 8. |

| | |
|---|---|
| *PALIN, A. G., B.M., B.Ch. Oxon. | Litfield House, Clifton Down, Bristol 8. |
| *PARKES, J. A., M.B., Ch.B. Liverp. | 8 South Road, Redland, Bristol 6. |
| *PARKES, P. W. J. | 164 Filton Avenue, Bristol 7. |
| PARKINSON, Lt.-Col. G. S., *D.S.O.* | London School of Hygiene and Tropical Medicine, Keppel Street, London, W.C. 1. |
| *PARRY, R. H., M.B., B.S.Lond. | Public Health Offices, Bristol 1. |
| PARSONS, Sir J. H., M.B., B.S., B.Sc. Lond. | 54 Queen Anne Street, London, W. |
| *PAUL, R. G. | 23 Pembroke Road, Bristol 8. |
| *PAULI, C. H. | Pagan's Hill Farm, Chew Stoke. |
| *PERKS, RUTH M.B., Ch.B. | Auckland House, Clifton Down. |
| *PERRY, B., M.D., Ch.B.Brist. | Canynge Hall, Clifton, Bristol 8. |
| *PETTY, W. J., M.B., B.S. | "Craigside," Worle, Weston-super-Mare. |
| PHELPS, P. C., M.B., Ch.B. | 23 St. Alban's Road, Bristol 6. |
| *PHILLIPS, P., M.B., Ch.B. Brist. | Southmead Hospital, Bristol. |
| *PIRRIE, A. L., M.B., Ch.B. Glasg. | 528 Kensington Hill, Bristol. |
| *POCOCK, J. A., M.A., M.B., B.Ch. Cantab. | Medical Postgraduate Dept., The University, Bristol. |
| *POND, MRS. M. H., M.B., B.Ch. Cantab. | 24 Cherrington Road, Henleaze, Bristol 7 |
| PORTER, C. P. | 28 Mill Street, Kidderminster. |
| *PORTER CHARLES, M.A. Oxon. | 124a Redland Road, Bristol 6. |
| *POTTER, MABEL F., M.D., Brist. | 19 Royal Park, Clifton, Bristol 8. |
| *POWELL, H. F., M.D. Brux. | Ellenborough Park, Weston-super-Mare. |
| *PRATT, W. W. | Feltham Farm, Pucklechurch. |
| *PRICE, ANNIE B., B.Sc., M.B., Ch.B. | Castleton Nursing Home, Bolters Lane, Banstead, Surrey. |
| *PRICE, C. H. G., M.B., Ch.B. Brist. | Claverham House, Claverham, Yatton. |
| *PRIDIE, K. H., M.B., B.S. Lond. | 58 St. John's Road, Clifton, Bristol 8. |
| *PRIDIE, MRS. KENNETH | 58 St. John's Road, Bristol 8. |
| *PROWSE, D. C., M.B., Ch.B. Brist. | Wigmore House, Thornbury, near Bristol. |

| | |
|---|---|
| *RANKIN, P. C., M.B., Ch.B. Glasg. | Lawrence Hill House, Bristol. |
| *RIDGWAY, J., M.B., Ch.B. | Hanham, Bristol. |
| *ROBERTS, J. A. F., M.A. Cantab., M.B., Ch.B., D.Sc. Edin. | Stoke Park Colony, Bristol. |
| *ROBERTS, J. A. L., M.B., Ch.B. | 75 High Street, Kingswood, Bristol. |
| *ROBERTSON, D., M.B., Ch.B. Edin. | 2 Knowle Road, Knowle, Bristol 4. |
| *ROBERTSON, L. G., M.B., Ch.B. | 162 Redland Road, Bristol 6. |
| *ROGERS, H., M.D. Brist. | 91 Old Park Ridings, Grange Park, N.21. |
| *ROLFE, R., B.A., M.B., B.C. Cantab. | Park House, St. Andrew's Road, Avonmouth. |
| *ROWLANDS, R. D. | Connaught Military Hospital, Knaphill, Surrey. |
| *ROWLEY, A. T. | 13 Walliscote Road, Weston-super-Mare. |
| *RUDOLF, G. DE M. | Seawalls, Clevedon. |
| *RYAN, P. B., M.B., Ch.B. | The Paddock, Frenchay. |

| | |
|---|---|
| *SAMPSON, O. E. L., M.B., Ch.B. Brist. | 77 Stackpool Road, Bristol 3. |
| *SCARFF, G., M.B., Ch.B. Edin. | 83 Pembroke Road, Clifton, Bristol 8. |
| *SHEPHERD, H. L., M.B., Ch.M. Brist. | 79 Pembroke Road, Clifton, Bristol 8. |
| *SHORE, T. L. H., M.A., M.B., B.Ch. | Linden Lodge, Haines Hill, Taunton. |
| *SHORT, A. R., M.D. Lond. | 69 Pembroke Road, Clifton, Bristol 8. |
| *SIMPSON, T. E. N. | "West Hythe," Old Patchway, Bristol. |
| *SMYTHE, H. J. D., *M.C.*, M.D., M.S. Lond. | Canynge Hall, Bristol 8. |
| *SNAWDON, J. W. E., M.B., Ch.B., B.D.S. | Litfield House, Clifton, Bristol 8. |
| *SOLTAU, H. K. V., M.D. Lond. | 19 The Avenue, Clifton, Bristol 8. |
| *SOMERS, MARY A. E. | 2 Ashcombe Road, Weston-super-Mare. |
| *SPARKS, J. V., B.A. | The Middle House, Felton, Som. |
| *SPOOR, A., M.B., Ch.B. Cantab. | The Hydro, Bristol 1. |
| *STEPHEN, R. J. | 92 Redland Road, Bristol 6. |
| *STROVER, H. W. M., *O.B.E.*, M.B., Ch.B. Aberd. | 90 Redland Road, Bristol 6. |
| *STRUTHERS, A. J., M.B., Ch.B. Glasg. | 100 Church Road, Redfield, Bristol 5. |
| *STRUTHERS, GEO., M.B., Ch.B. Glasgow | 100 Church Road, Redfield, Bristol 5. |
| *SUTTON, G. E. F., M.D. Lond. | 1 Pembroke Road, Clifton, Bristol 8. |
| *SYMES, J. O., M.D. Lond. | 6 Pembroke Vale, Clifton, Bristol 8. |

| | |
|---|---|
| *TALLACK, MAJOR R. J. K., R.A.M.C. .. .. | (Kenya) Field Amb., A.P.O., E.A. Command. |
| *TASKER, D. G. C., M.B., M.S. Lond. .. .. | 9 College Fields, Clifton, Bristol 8. |
| *TAYLOR, A. L., M.D. Lond. .. .. .. .. | The General Hospital, Bristol 1. |
| TAYLOR, A. L. .. .. .. .. .. .. .. .. .. | The Royal Infirmary Bristol 2. |
| THIN, J. .. .. .. .. .. .. .. .. .. .. .. | 54 and 55 South Bridge, Edinburgh. |
| *TODD, A. T., *O.B.E.*, M.D. Edin. .. .. .. | Royal Park House, Clifton, Bristol 8. |
| *TOMLINSON, STERLING, M.B., B.Ch. .. .. | Clouds Hill House, St. George, Bristol 5. |
| *TRIBE, WINIFRED N., M.B., Ch.B. .. .. .. | Dings House, St. Philip's, Bristol 2. |
| TRINICK, RICHARD H. .. .. .. .. .. .. .. | 42 Cherry Valley Gardens, Belfast. |
| TRIPP, DOROTHY M. H. .. .. .. .. .. .. | Mission Hospital, Karim Nagar, Nizam's Dominions, South India. |
| *TRYON, VICTORIA S., M.B., Ch.B. Brist. .. | 3 Harley Place, Clifton, Bristol 8 |
| *WAGSTAFFE, E. M., M.B., Ch.B. .. .. .. | Winford Orthopædic Hospital. |
| *WANSBROUGH, T. B., M.B., Ch.B. Brist. .. | 23 Pembroke Road, Clifton, Bristol 8. |
| *WALKER, CYRIL H., M.B. Cantab. .. .. .. | Harewood, Clapton-in-Gordano, Som. |
| WAKE, S. C., M.B., Ch.B. Brist. .. .. .. | "Stanhope," Bideford, N. Devon. |
| *WARD, H. W. .. .. .. .. .. .. .. .. .. | Chipping Manor, Wotton-under-Edge. |
| *WARREN, R., M.A., M.D. Oxon. .. .. .. | Heathgates, Weston-super-Mare. |
| *WATSON-WILLIAMS, E., *M.C.*, M.B. Cantab., Ch.M. Brist. | 65 Pembroke Road, Clifton, Bristol 8. |
| WATTS, J. B. V. .. .. .. .. .. .. .. .. | Frantzina's Rust, Barberton, Transvaal, S. Africa. |
| WEDDELL, C. W. .. .. .. .. .. .. .. .. | Boydville, 46 Miller Street, West Melbourne, Australia. |
| *WEEKS, A. .. .. .. .. .. .. .. .. .. .. | 39 Badminton Road, Downend. |
| *WHITE, R. F., M.B., Ch.B. .. .. .. .. .. | "Wellclose," High Street, Nailsea. |
| *WIGODER, SYLVIA, M.D., Ph.D. .. .. .. | |
| *WILBOND, R. G. .. .. .. .. .. .. .. .. | 76 Chesterfield Road, St. Andrew's Pk., Bristol. |
| *WILLIAMS, I., M.B., Ch.B. Brist. .. .. .. | "Ardrem," Hill View, Henleaze, Bristol. |
| *WILLIAMS, S. R., M.B. Lond. .. .. .. .. | Buckfastleigh, Devon. |
| *WILLOUGHBY, J., M.B., B.Ch. .. .. .. .. | "Kingsholme," Clevedon Road, Nailsea. |
| *WOOD, D. .. .. .. .. .. .. .. .. .. .. | 105 Pembroke Road, Clifton, Bristol 8. |
| *WOOD, URSULA, M.B., Ch.B. .. .. .. .. | Henley Lodge, Yatton, Som. |
| *WOOD, W. V., *M.C.*, B.A. Cantab. .. .. .. | Henley Lodge, Yatton, Som. |
| *WOODMAN, DOROTHY, M.D. Lond. .. .. .. | 10 Tyndall's Park Road, Clifton, Bristol 8. |
| *WOOLLEY, W., M.B., Ch.B. Brist. .. .. .. | 239 Cranbrook Road, Redland. |
| *WRIGHT, A. J. M., M.B., B.S. Lond. .. .. | Harley Cottage, Clifton Park, Clifton, Bristol 8 |
| *WRIGHT, WINIFRED M., M.B., B.S. Lond. .. | Merrymead, Shepton Mallet, Som. |
| *ZEALAND, L., M.D. Man. .. .. .. .. .. .. | Brecon Lodge, Westbury-on-Trym, Bristol. |

